# Analysing the outbreaks of leptospirosis after floods in Kerala, India

**DOI:** 10.1186/s12942-024-00372-9

**Published:** 2024-05-13

**Authors:** Oluwafemi John Ifejube, Sekhar L. Kuriakose, T. S. Anish, Cees van Westen, Justine I. Blanford

**Affiliations:** 1 Geo-Information Science and Earth Observation (ITC), University of Twente, Enschede, The Netherlands; 2 Kerala State Disaster Management Authority (KSDMA), Thiruvananthapuram, Kerala India; 3 Government Medical College, Malappuram, Kerala India

**Keywords:** Climate, Disaster, Flood, Epidemiology, Leptospirosis, Assessment, Incidence, Outbreak

## Abstract

A growing number of studies have linked the incidence of leptospirosis with the occurrence of flood events. Nevertheless, the interaction between flood and leptospirosis has not been extensively studied to understand the influence of flood attributes in inducing new cases. This study reviews leptospirosis cases in relation to multiple flood occurrences in Kerala, India. Leptospirosis data were obtained for three years: 2017 (non-flood year) and two years with flooding—2018 (heavy flooding) and 2019 (moderate flooding). We considered the severity of flood events using the discharge, duration and extent of each flooding event and compared them with the leptospirosis cases. The distribution of cases regarding flood discharge and duration was assessed through descriptive and spatiotemporal analyses, respectively. Furthermore, cluster analyses and spatial regression were completed to ascertain the relationship between flood extent and the postflood cases. This study found that postflood cases of leptospirosis can be associated with flood events in space and time. The total cases in both 2018 and 2019 increased in the post-flood phase, with the increase in 2018 being more evident. Unlike the 2019 flood, the flood of 2018 is a significant spatial indicator for postflood cases. Our study shows that flooding leads to an increase in leptospirosis cases, and there is stronger evidence for increased leptospirosis cases after a heavy flood event than after a moderate flooding event. Flood duration may be the most important factor in determining the increase in leptospirosis infections.

**Supplementary Information:**

The online version contains supplementary material available at 10.1186/s12942-024-00372-9.

## Introduction

More than 2 billion people have been affected by floods in the past two decades [1] and there is clear evidence that the number of flood incidences is increasing due to climate change [2, 3]. The effects of flooding on health are varied and include a range of rodent-borne, water-borne, and vector-borne diseases [4]. One of these water-borne diseases associated with flooding is leptospirosis [4–6]. Severe flooding has led to a higher number of infections in areas endemic to leptospirosis [7, 8].

The global burden of leptospirosis was estimated to be about 2.90 million Disability Adjusted Life Years (DALYs), with most coming from tropical LMICs [9]. Due to the close resemblance of symptoms with other acute febrile illnesses, clinical diagnosis is often missed or delayed leading to severe complications and increased mortality [10]. Despite the variability in the quality of disease incidence reporting, 59,000 persons are estimated to die every year from leptospirosis [9]. The case fatality of leptospirosis can be as low as 6% or as high as 50% depending on the availability of supportive care [11].

Human infections of leptospirosis are caused by either direct contact with the urine of an infected animal or more usually, indirect exposure from a contaminated environment such as water and soil (that have been contaminated by the urine of an infected host) [12]. The incubation period for leptospirosis can range from 2 to 20 days after initial exposure to the bacteria with infections lasting for weeks to months [13]. Symptoms include fever, headache, and myalgia [12].

Several risk factors have been identified and are associated with the occurrence of leptospirosis [14, 15]. In LMICs, higher chances of direct and indirect human infections have been found to occur due to occupational affiliations (e.g., abattoirs, livestock, and agricultural farm workers) [12], as well as poor sanitation and hygiene practices, and rodent density in the environment [16]. The climatic environment also plays a complex role in the interactions between humans, zoonotic hosts, and pathogens in the environment [17]. Heavy rainfall and flooding have been shown to contribute to an increase in leptospirosis infections [18].

The use of spatial and temporal analytical methods has substantially improved our understanding of leptospirosis epidemiology [19]. Several studies have connected the incidence of leptospirosis with flooding by spatial and temporal analysis [7, 8, 20, 21]. Nevertheless, limited studies have related and compared flood events and leptospirosis incidence across multiple years. Since not all flood events in endemic regions lead to an outbreak [21], there is a need to study the extent of the influence of flood characteristics in inducing leptospirosis infections by comparing multiple flood events. The impact of climatic changes on human health can be more accurately assessed using geospatial tools and techniques [22]. The analysis of the dynamics between flood exposure and leptospirosis infection can be used to further understand the transmission pattern of infection during floods.

The purpose of this study was to (i) explore the spatial distribution of leptospirosis cases in relation to flooding and (ii) examine the relationship between leptospirosis incidence and flood events in Kerala. To achieve this, we retrospectively examined the cases of leptospirosis across flood phases (before, during and after) and across three consecutive years that included a non-flood year, a severe flood year and a less severe flood year.

### Study area

With a population of 33 million people, Kerala state is located on the southwestern coast of India. Nearly all districts in Kerala are vulnerable to multiple hazards, however, flooding stands out as the most common and may yet become an annual affair in Kerala [23]. In 2018, Kerala experienced a severe flood, largely due to an unexpected amount of rainfall in the monsoon season [24]. The 2018 flood was the most extreme in Kerala in almost a century, nevertheless, other flood events occurred in consecutive years [25].

Although the cases of leptospirosis in Kerala were first reported three decades ago, the floods of 2018 revitalized their presence [26, 27]. Leptospirosis is endemic in Kerala with the highest mortality rates recorded in comparison to other infectious diseases [28]. In 2018, the highest number of cases were reported in the southern districts of Kerala [29]. For this reason, two of the most affected districts by leptospirosis (Alappuzha and Pathanamthitta) were selected to better understand the risk of leptospirosis incidences in relation to flooding events.

**Fig. 1:**
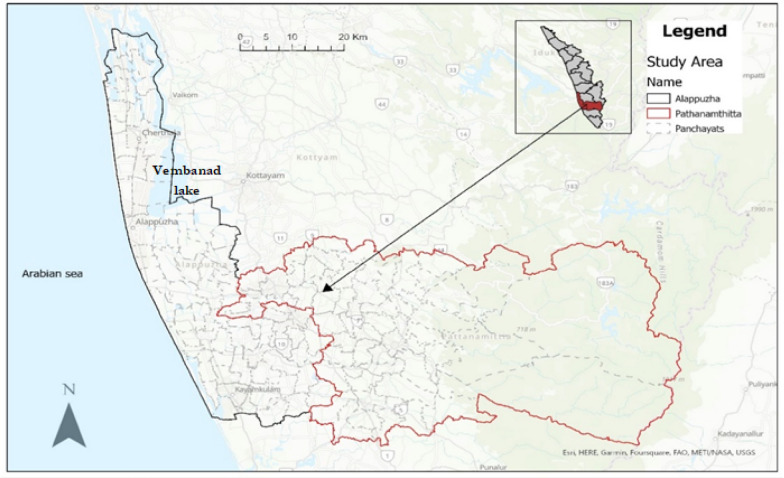
Study area and the panchayats

The districts—Alappuzha and Pathanamthitta consist of 135 local administrative units called panchayats (Fig. 1) home to a total population of 3.3 million people [30]. There are distinctions between the two districts geographically. Alappuzha is the smaller of the two districts with a size of 1414 sq. km. Pathanamthitta is almost double the size of Alappuzha covering 2653 sq. km. According to the District Planning Offices (DPO), Alappuzha has a higher population per sq. km (1079) than Pathanamthitta (453) [31].

Alappuzha is a low-lying area with some parts below sea level and is located between the Vembanad Lake and the Arabian Sea. Pathanamthitta is highly vegetated with forest reserves that account for 50% of the district area [31] and a varied topography with mountainous regions in the east. On average temperatures are 27 °C in Alappuzha and 25 °C in Pathanamthitta with the rainy season occurring during the annual southwest monsoon period (June–September).

## Methods

We examined the relationship between flooding and leptospirosis in the study area for three years: 2017(a non-flood year), 2018 (a severe flood year) and 2019 (a less severe flood year). The flood events in 2018 and 2019 were assessed with respect to flood characteristics (e.g., discharge, duration and extent) and the impact this had in the study area. Furthermore, the distribution of cases regarding the flooding events was assessed spatially and temporally. Statistical comparisons were made between leptospirosis cases across each year and within each year to ascertain the relationship between flooding and leptospirosis.

Data was collected for the 3 years (2017 to 2019). For this study’s purpose, three phases of flooding were defined to examine how cases of Leptospirosis changed over time and space. The phases are the periods before the flood, during the flood, and after the flood. The associated dates for each phase are defined in Table 1 and coincide with the reports provided by the Government of Kerala. No reported flood events occurred in 2017. The 2018 floods occurred due to heavy rainfall within the monsoon period [32]. In this study, the 2018 flood period was defined based on the flood maps published by [32]. The 2019 flood period was in August 2019 [33].

**Table 1:** Classification of flood phases used for 2017–2019

Year	Preflood (3 months before)	Flood period (during)	Post-flood (3 months after)
2017	No flood	No flood	No flood
2018	April 15–July 15	July 16–August 28	August 29–November 29
2019	May 7–August 7	August 8–31	September 1–December 1

For all three years, the pre-flood phase is defined as three months before the flood, and the post-flood phase as three months after the flood. Three months were chosen because it provides sufficient time for incubation and transmission between hosts [8].

All data used in this study are described next and summarised in Table 2. The datasets used include the reported cases of leptospirosis, the variables relevant to the flood events, and the population residing in the study area. Data on Leptospirosis cases were obtained from KSDMA for three years (2017–2019). The daily reported cases were aggregated for each panchayat and each epidemiological week (epi week), where epi week 1 starts on the first Sunday of the new year and ends on the Saturday of the same week [34]. The administrative boundaries of each panchayat in the study area were obtained from KSDMA.

**Table 2:** Data used in this study

Datasets	Description^a^	Type (resolution)	Date	Source
Leptospirosis cases	Daily records of positive leptospirosis cases in 2017, 2018 and 2019 at the panchayat level	Vector file (point)	2019	KSDMA
Administrative boundaries	Boundaries of Kerala districts and panchayats in the study area. The total population for each panchayat is included	Vector file (polygon)	2022	KSDMA
Flood extent	The geographical extent of the flood of 2018 and 2019	Raster file (30 m)	2023	[35]
River discharge	The daily amount of water discharges in river channels obtained for 2018 and 2019	Excel file	2023	KSDMA
Precipitation	Daily rainfall for Alappuzha for 2018 recorded at local stations	Excel file	2022	KSDMA
	Daily rainfall from global climatic data for the study area from 2017 to 2019	Excel	2022	ERA5
Population density	Distribution of the human population in the study area	Raster file (30 m)	2021	Meta

^a^Daily records of a year refer to the 1st of January till the 31st of December for the given year

Precipitation data was obtained from local and global sources. Daily rainfall for 2018 was provided by KSDMA only for the rainfall station in Alappuzha. To capture rainfall for the study area, ERA5 daily precipitation data for 2018 were obtained. ERA5 is the fifth generation of global atmospheric reanalysis by the European Centre for Medium-Range Weather Forecasts (ECMWF) and has been used in other studies [36–38]. ERA5 data were validated by comparing the rainfall data obtained for the station in Alappuzha with the ERA5 data. A 0.67 correlation was found (Supplementary Information 1). Based on the classification of the correlation by [39] and validation of the aforementioned studies, we considered it acceptable to use ERA5 data to represent precipitation for the study area. The ERA5 daily precipitation was therefore used for all three years (2017–2019) in both districts.

River discharge data were obtained from daily river discharges to capture the flood occurrence in 2018 and 2019. Data were measured at two stations in the study area: Station Q_Erapphuza (located in the Alappuzha district), and Station Q_Kurudamannil (located in the Pathanamthitta district). A total number of 430 (out of 1460) days were missing (424 for Q_Erapphuza and 6 for Q_Kurudamannil) in the 2018 river discharge data. Missing values were infilled using temporal trend analysis [40] (the summary of the estimation is provided in Supplementary Information 2). This estimation uses the time-series values of consecutive days using the Interpolated Univariate Spline method in the SciPy Interpolation package [40]. To match the leptospirosis case data, river discharge data was averaged by epi week for each year.

Flood extents during 2018 and 2019 were captured by raster maps created by [35] from Sentinel-1 Radar imagery. The raster maps were obtained by determining the difference in the amount of water bodies in the flood phase as compared to the preflood phase. These maps were used to demarcate the extent of flooding in Kerala during the flood phases for both years.

The population density was obtained from Meta [41] using the most recently available data (2021) for the study area. Meta’s population maps have been used in a variety of studies (e.g., [42–44]) and are considered representative. Essentially, the population density maps were created by identifying human-made buildings from high-resolution satellite imagery and assigning population estimates using convolutional neural network (CNN) architectures integrated with census data [41]. The final population map provides the distributed population density raster at a 30m resolution, thereby enabling accurate population estimates to be determined in rural areas [41].

### Characterization of flood events

Descriptive statistics were used to evaluate the amount of precipitation that occurred and how this coincided with the amount of river discharge for each year. The extents of the flood event were assessed for each year. The flood's impact was assessed for each flood year (2018 and 2019) by determining the percentage of panchayats and the population exposed during each flooding event. All spatial analyses were performed in ArcGIS Pro version 3.1.1.

### Spatiotemporal distribution of leptospirosis across years

The total number of cases of leptospirosis and river discharges in each of the three years (2017–2019) were cross-examined through boxplots. An epidemiological curve was constructed to compare the trend of river discharge with the number of cases. The cases during the two flooded years were compared against 2017 (non-flood year) to understand their differences and similarities in order to understand the role flooding has on the occurrence of leptospirosis infections within the population.

### Spatiotemporal distribution of leptospirosis across flood phases

The occurrence of leptospirosis cases was analyzed for each flood phase of the flooded years (2018 and 2019). Cluster and outlier analyses were conducted using the Anselin Local Moran's I statistic [45] to identify potential leptospirosis hotspots (high-high clusters: where panchayats with high leptospirosis incidence are near other panchayats with high leptospirosis incidence), cold spots (low-low clusters: panchayats with low leptospirosis incidence are near other panchayats with low leptospirosis incidence), and spatial outliers (high-low and low–high clusters: panchayats with high incidence surrounded by areas with low incidence and vice versa) among the panchayats. The flood-induced incidence rates were computed using the population exposed during floods and the post-flood cases. The incidence rates of leptospirosis were calculated by dividing the total number of post-flood cases by the total population affected and expressed as incidence per 100,000 people.

### Comparison of the relationship between flood and leptospirosis

Spatial variation in leptospirosis cases and flooding was compared using a regression analysis. The regression between flood extent and cases in 2018 was compared against the flood extent and cases in 2019 to determine if there are differences between these two events. The spatially varying coefficient (SVC) regression model (in Eq. 1) was employed to compare the relationship between each pair (flood extent and the number of cases) for the flooded years (2018 & 2019). The R software was used to perform this regression analysis. Source code is provided in Supplementary Information 3.

1 $${\text{y}}({\text{s}})={\beta }_{0}\left(s\right)+ {\beta }_{1}{x}_{1}\left(s\right),$$

where y is the total number of leptospirosis cases, s refers to the locations of each panchayat. ${\beta }_{0},{\beta }_{1}$ are the regression coefficients, ${x}_{1}$ refers to the flood extent.

## Results

### Characterization of flood events

**Fig. 2:**
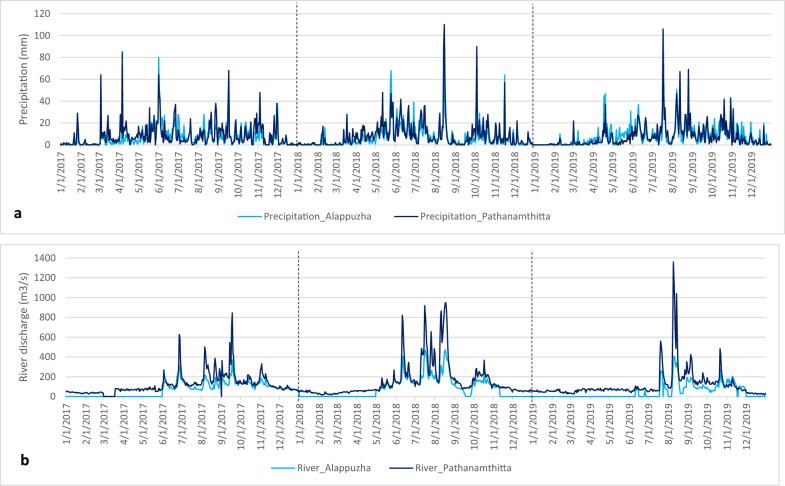
Time series plot of precipitation and river discharge from 2017 to 2019 (**a**) precipitation (**b**) river discharge (where the light blue lines represent Alappuzha and the thick blue lines represent Pathanamthitta)

Table 3 summarizes the precipitation for the two districts and the three time periods for 2017–2019. A higher amount of precipitation was recorded in Pathanamthitta (2788–3144 mm) than in Alappuzha (2602–2650 mm). In the three years, rainfall was highest in Pathanamthitta during 2018 (3144 mm), highest in Alappuzha during 2017 (2650 mm) and lowest in both districts during 2019 (2602 mm in Alappuzha and 2788 mm in Pathanamthitta) (Table 3). Unlike during 2017, increased and longer rainfall occurred majorly in the southwest monsoon period during 2018 and 2019 (Fig. 2).

**Table 3:** Total precipitation and maximum river discharge observed between 2017 and 2019

	Category	2017	2018	2019
Alappuzha	Total precipitation (mm)	2650	2602	2602
	Maximum river discharge (m^3^/s)	372.9	472.2	409.9
Pathanamthitta	Total precipitation (mm)	3086	3144	2788
	Maximum river discharge (m^3^/s)	846.6	949.9	1360.3

In all three years, the maximum river discharge was consistently higher in Pathanamthitta (ranging from 846.6 to 1360.3 m^3^/s) than in Alappuzha (ranging from 372.9 to 472.2 m^3^/s) (Table 3; Fig. 2). River discharge was highest in Alappuzha during 2018 (max river discharge 472.2 m^3^/s), highest in Pathanamthitta during 2019 (max river discharge 1360.3 m^3^/s), and lowest in both districts during 2017 (max river discharge: 372.9 m^3^/s in Alappuzha and 846.6 m^3^/s in Pathanamthitta) (Table 3).

**Fig. 3:**
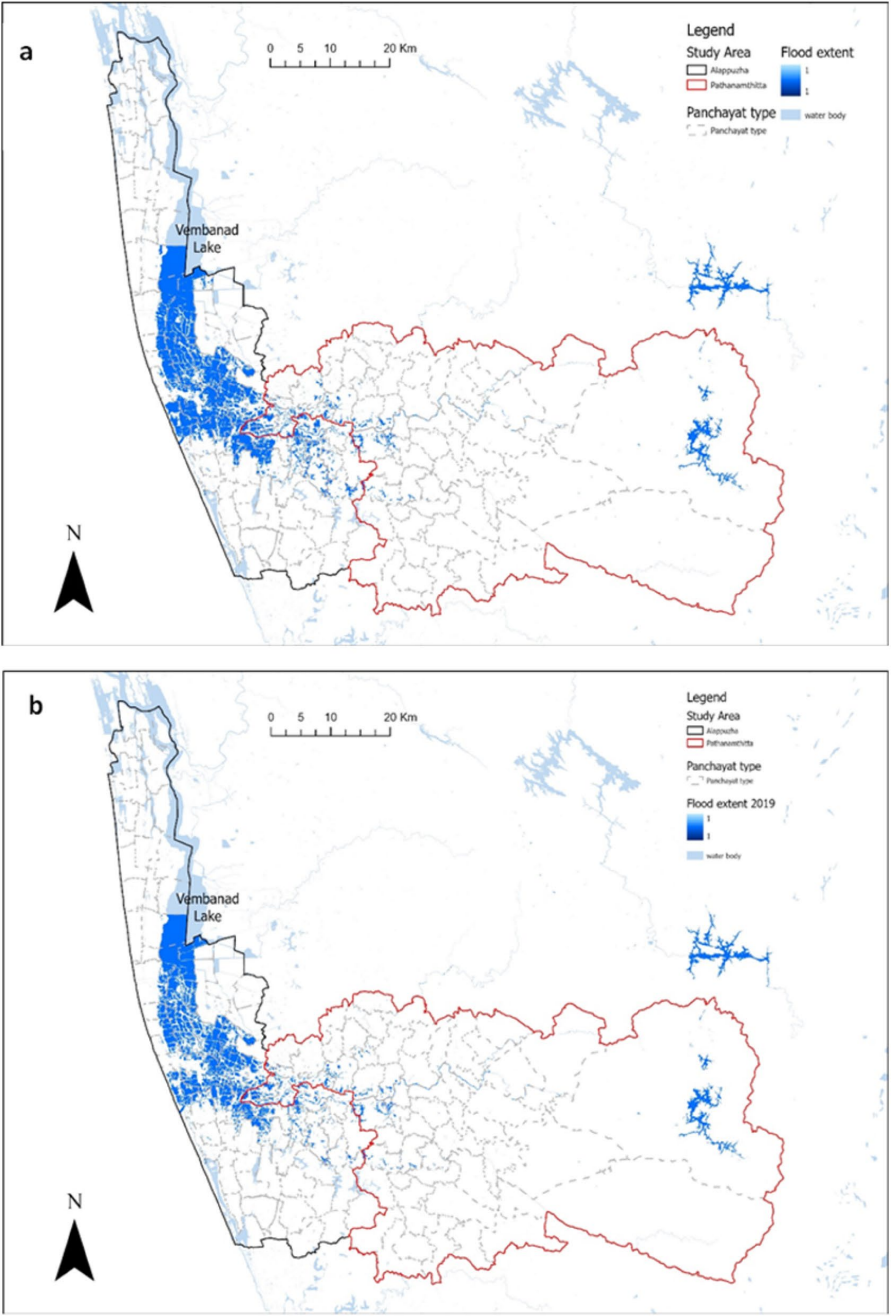
Flood extent maps in the study area (**a**) 2018 (**b**) 2019

The panchayats in the central areas of Alappuzha were the most affected by floods (Fig. 3). The Vembanad Lake is the largest water body close to flooded areas in Alappuzha. Despite high river discharges in Pathanamthitta, the flood extent was smaller than that of Alappuzha.

Table 4 shows the total area affected by the flood during 2018 and 2019 was 278 km^2^ and 220.0 km^2^, respectively. A total of 81 panchayats were exposed to flood in both years but the number of panchayats exposed to floods in Alappuzha was consistently higher than that of Pathanamthitta. An equal portion of panchayats (60%) were exposed to floods in 2018 and 2019; however, the population exposed in 2019 (1.0%) was lower than that of 2018 (1.9%). The population exposed to the flood was higher in Alappuzha than in Pathanamthitta.

**Table 4:** Area, count of panchayats and population exposed to floods in 2018 and 2019

Year	2018			2019		
Categories	Alappuzha (%)	Pathanamthitta (%)	Total (%)	Alappuzha (%)	Pathanamthitta (%)	Total (%)
Area flooded (km^2^)	238 (19.2)	40 (1.5)	278 (9.5)	184 (14.9)	36 (1.4)	220 (5.8)
Panchayats (N = 135)	43 (31.9)	38 (28.1)	81 (60)	42 (31.3)	39 (28.9)	81 (60)
Population (N = 3,193,446)	53,145 (1.7)	5990 (0.2)	59135 (1.9)	29,060 (0.9)	3804 (0.1)	32,864 (1.0)

In 2018, the peak in river discharge occurred on the 18th of August 2018 (949.9m^3^/s). At a similar time of the year in 2019, the river discharge peaked on the 8th of August 2019 (1360.3m^3^/s) (Fig. 2). Although the highest river discharge occurred in 2019, a longer period of discharge occurred in 2018 (Fig. 2).

### Spatiotemporal distribution of leptospirosis across years

**Fig. 4:**
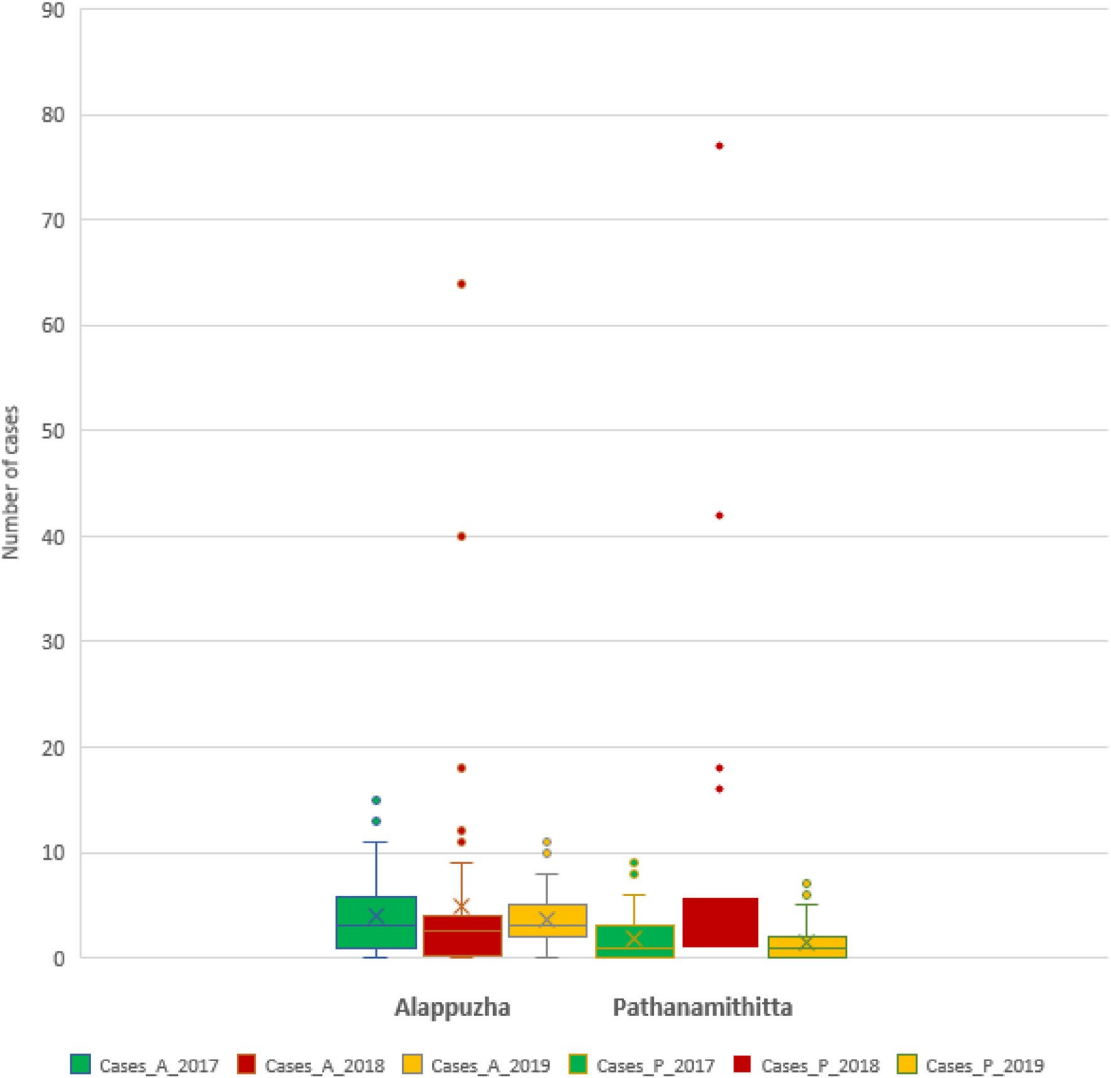
Total number of incidences of leptospirosis between 2017 and 2019 (A boxplot where green boxes = cases in 2017; red boxes = cases in 2018; yellow boxes = cases in 2019. A = Alappuzha, P = Pathanamthitta)

**Fig. 5:**
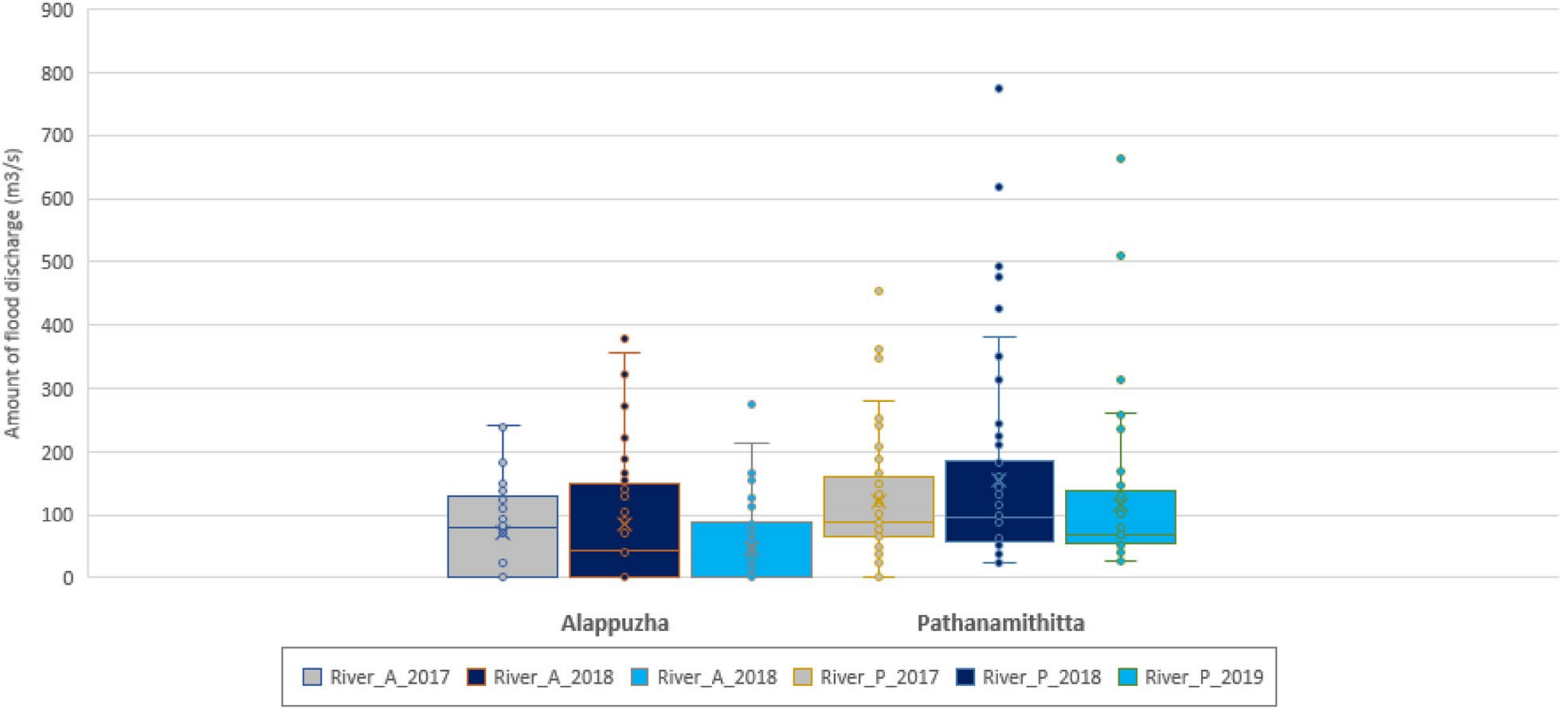
Amount of river discharge in study per epi week (where grey boxes = river discharge in 2017, dark blue boxes = river discharge in 2018 and light blue boxes = river discharge in 2019. A = Alappuzha, P = Pathanamthitta)

The results of descriptive analyses of leptospirosis cases and river discharge are shown in Table 5. Leptospirosis cases were reported for all three years with the highest number of cases occurring in 2018. Except for 2018, more cases were reported in Alappuzha than in Pathanamthitta. The highest reported cases per epiweek during 2018 in both districts were at least four times higher than during other years (Table 5; Fig. 4). Despite the high river discharges that occurred in 2018 and 2019, only the cases in 2018 showed a high increase (Fig. 5).

**Table 5:** Summary of leptospirosis cases and average river discharge between 2017 and 2019 in the study area

	Leptospirosis cases						River discharge (m^3^/s)					
District	Alappuzha			Pathanamthitta			Alappuzha			Pathanamthitta		
Category	2017	2018	2019	2017	2018	2019	2017	2018	2019	2017	2018	2019
Mean	3.9	4.9	3.6	1.9	6	1.5	71.1	84.4	46	120.7	154.9	115.1
Standard Deviation	3.6	10.4	2.7	2.5	12.1	1.7	9.5	102.4	66.9	12.9	160.9	117.1
Minimum	0	0	0	0	0	0	0	0	0	0	236	26.7
Maximum	15	64	11	9	77	7	242.2	379.4	275.5	454.1	774.3	664
Sum	204	253	185	98	312	77	3694.7	4389.2	2393.1	6278.7	8056.3	5984

**Fig. 6:**
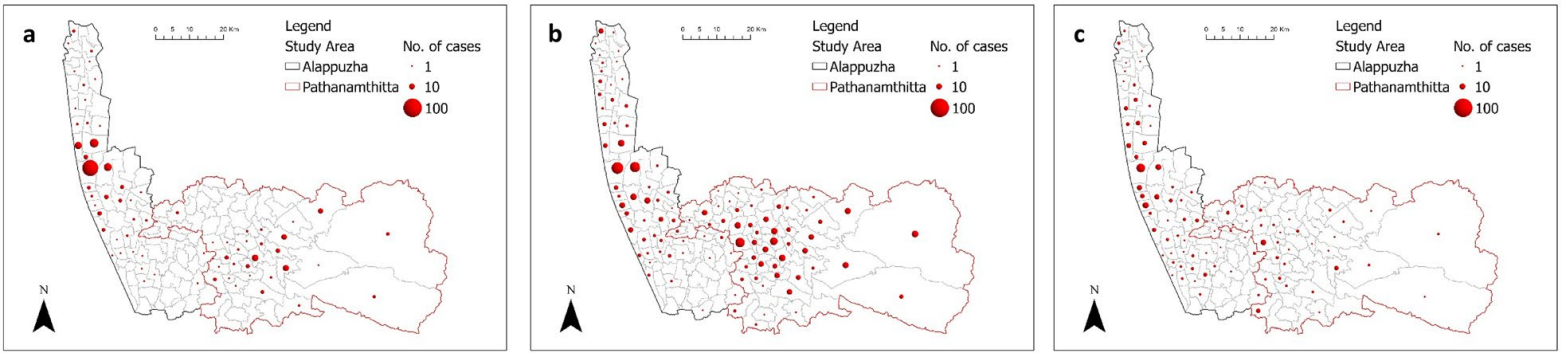
Spatial distribution of the total number of cases by panchayats in (**a**) 2017 (**b**) 2018 (**c**) 2019

Leptospirosis cases were observed in multiple panchayats and the distribution of leptospirosis cases varied over the three years. The total number of panchayats reporting at least 1 case of Leptospirosis rose from 68 in 2017 to 105 in 2018 and reduced again to 89 in 2019. Figure 6 shows that higher cases occurred in Alappuzha communities during 2017 and 2019. During 2018, the central parts of both districts were affected.

Significant clusters of leptospirosis cases were found for all three years (Supplementary Information 4). The cases in both flooded years (2018 and 2019) are therefore classified into the three different flood phases in further analyses.

### Spatiotemporal distribution of leptospirosis across flood phases

**Fig. 7:**
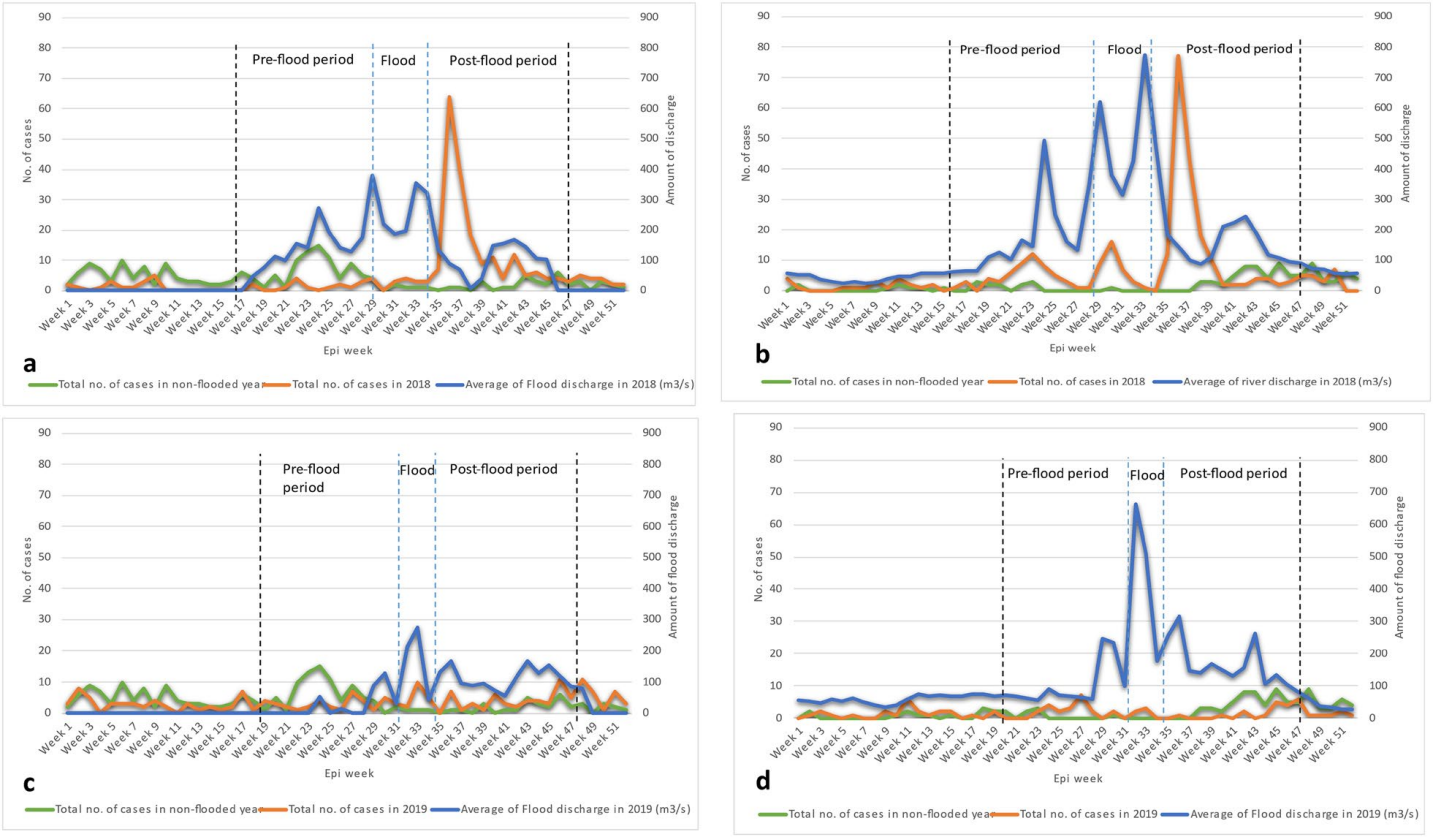
Time series plot of leptospirosis across flood phases in the study area. **a** Alappuzha in 2018. **b** Pathanamthitta in 2018. **c** Alappuzha in 2019. **d** Pathanamthitta in 2019

Figure 7 displays the differences between the trend of leptospirosis cases during the three flood phases of flooded years (2018 and 2019) and the non-flooded year (2017) in the study area. A sharp increase in the number of cases can be seen after the flood phase in both districts in 2018. The highest number of cases was reported during week 36, precisely on the 4th of September 2018, which is 17 days after the flood peak (18th of August 2018). For 2019 no major peaks were observed in either district. Leptospirosis cases in 2019 were found to be similar to or less than those reported in the non-flooded year.

**Fig. 8:**
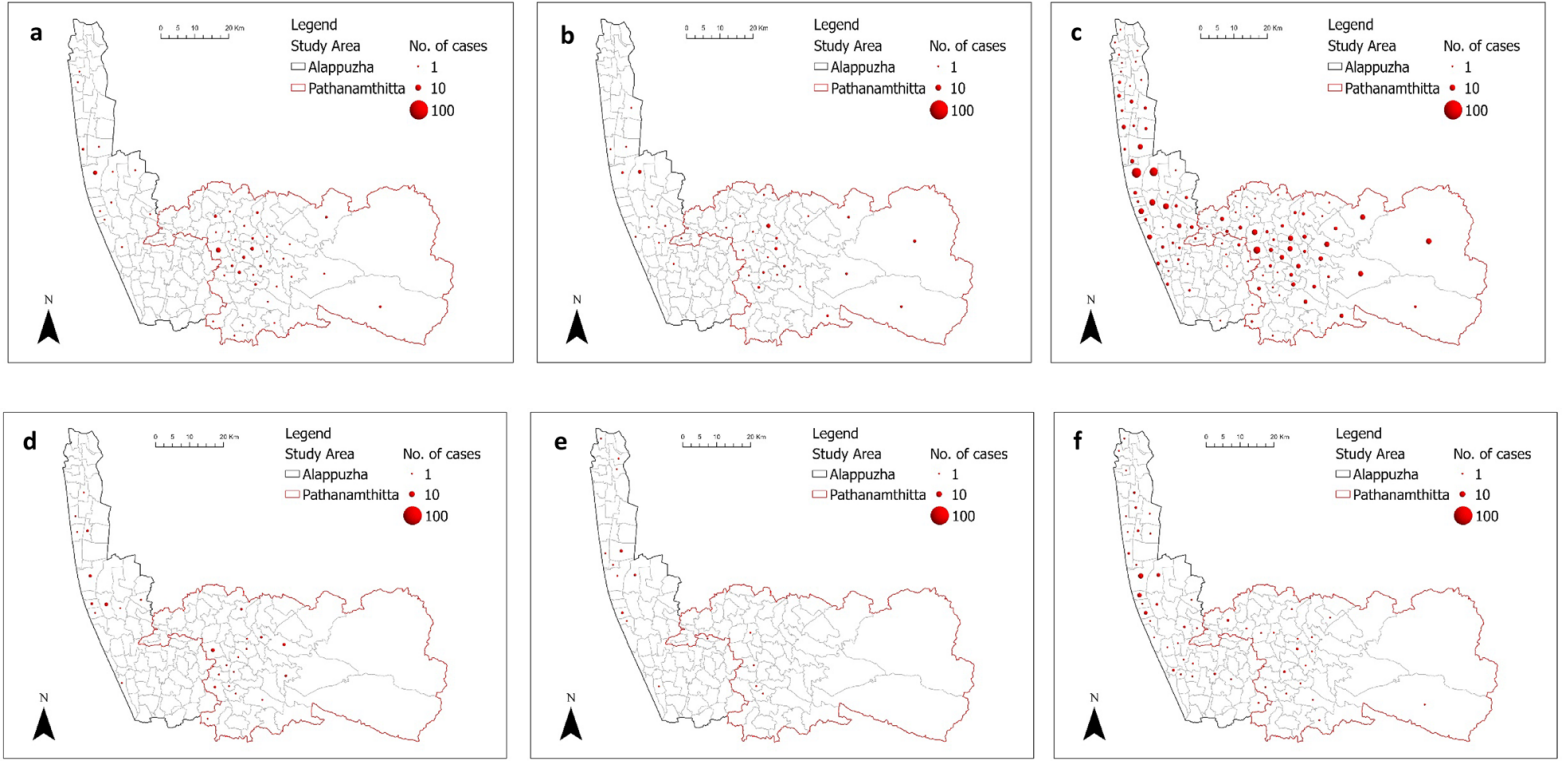
Spatial distribution of leptospirosis cases among panchayats across flood phases, **a** before the 2018 flood, **b** during the 2018 flood, **c** after the 2018 flood, **d** before the 2019 flood, **e** during the 2019 flood, **f** after the 2019 flood

The highest number of cases in 2018 occurred during the post-flood phase in both districts, but this was not during 2019 (Table 6a). The distribution of cases during the post-flood phase of 2018 is more evident than that of 2019 in both districts, even though the cases in all phases are distributed in similar locations (central parts of Alappuzha and Pathanamthitta) (Fig. 8). The number of cases during the flood phase was lower in comparison with other phases in both years.

**Table 6:** River discharge, leptospirosis cases across flood phases, and flood-induced incident rates

Phase	Category	2018		2019	
		Alappuzha	Pathanamthitta	Alappuzha	Pathanamthitta
(a) River discharge and leptospirosis cases across flood phases					
Pre-flood	Maximum river discharge (m^3^/s)	118.9	171.2	21.7	94.4
	Total leptospirosis cases	17	56	18	25
Flood	Maximum river discharge (m^3^/s)	277.9	498.3	138.0	341.7
	Total leptospirosis cases	15	36	16	4
Post-flood	Maximum river discharge (m^3^/s)	90.5	146.1	109.2	150.2
	Total leptospirosis cases	192	187	59	21
(b) Incidence rates from flooding in 2018 and 2019					
Post-flood	Population exposed to flood	53,145	5990	29,060	3804
	Incidence Rate per 100,000	361	3122	203	552

Table 6 shows the summary of river discharge and cases across the flood phases in the study area. The average amount of river discharge was highest during the flood phase in 2018 (277.9 m^3^/s in Alappuzha; 498.3 m^3^/s in Pathanamthitta) and 2019 (138.0 m^3^/s in Alappuzha; 341.7 in Pathanamthitta). The river discharges were consistently higher in 2018 than in 2019 in all phases, except during the postflood phase of 2019 (109.2 m^3^/s in Alappuzha; 150.2 m^3^/s in Pathanamthitta).

**Fig. 9:**
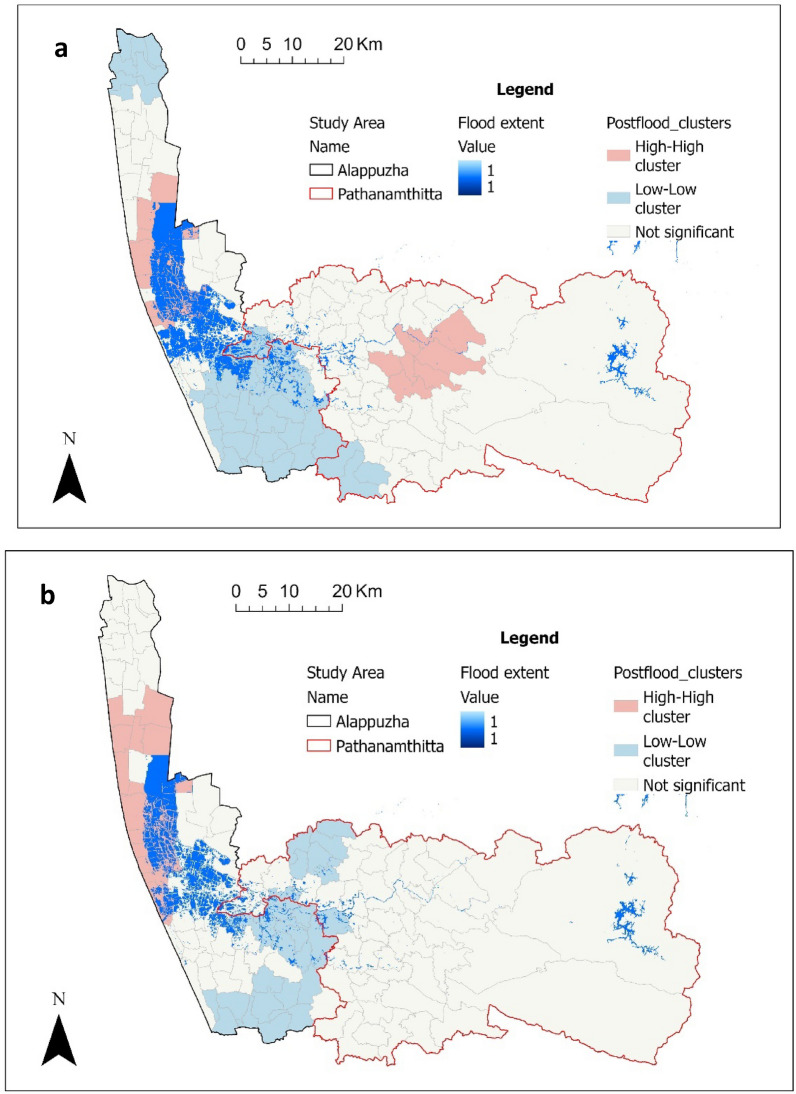
Flood extent and the cluster of post-flood incidences in (**a**) 2018 and (**b**) 2019

The clusters and outliers of leptospirosis cases across flood phases are presented in Supplementary Information 5. The result of overlaying the flood extent and the clusters of leptospirosis is shown in Fig. 9. It can be seen that the flood events in 2018 and 2019 are related to the hotspots (high–high clusters) of the case during the postflood phase. Using the population exposed during floods and the post-flood cases, the flood-induced incidence rates are computed. Higher flood-induced incidence rates per 100,000 were observed in 2018, and in Pathanamthitta (Table 6b).

### Comparison of the relationship between flood and leptospirosis

The result of SVC regression analyses is presented in Table 7. The flood extent of 2018 was found to be a significant explanatory variable for the 2018 post-flood leptospirosis cases at a 95% confidence level. Using the same confidence level, the flood extent of 2019 was not found to show statistical significance to post-flood leptospirosis cases in 2019.

**Table 7:** Spatial regression analysis for flood extents and post-flood cases

	Estimate	Std. error	Z value	Estimate	Std. error	Z value
	2018 Analysis (R^2^ = 0.978)			2019 Analysis (R^2^ = 0.825)		
(Intercept)	2.5943	0.9510	2.728**	0.4334	0.1080	4.015***
flood	1.1488	0.5319	2.160*	0.1475	0.2772	0.532

***p < 0.001, **p < 0.01, *p < 0.05

## Discussion

### Impact of flood events

The impact of the flooding events was analyzed based on the discharge, duration, and geographic extent of the flood. In all three attributes, the 2018 flooding event was more severe than that of 2019. As Pathanamthitta is located upstream of Alappuzha, it was the extreme rainfall in 2018 that caused the major flooding in both districts. This result is consistent with previous studies that have performed impact analyses for 2018 and 2019 flooding events [25, 46] in this study area, especially in central Alappuzha. Even though Alappuzha would have been more flooded than Pathanamthitta, we didn’t have reliable flood discharge data to demonstrate this. The flooding appears to have been aggravated by the Vembanad lake and other main rivers in Alappuzha. Although the spatial extents of both floods were similar, many more people were exposed to the 2018 flooding event.

### Interactions between flood and leptospirosis

The trend of leptospirosis during the flood periods offers invaluable insight into the dynamics between flood and leptospirosis in Kerala. The peak of leptospirosis cases occurred 17 days after the peak of the floods. This result is consistent with the findings by Sykes et al. that cases occur 2–20 days after initial exposure to the bacteria. It should be noted that the infections may have occurred on earlier dates because only the information on the reporting date at the facility was provided. The similarities in the trend of leptospirosis before the flood phase across the years suggest the presence of seasonal patterns of infection in the study area. The endemicity of leptospirosis around water bodies can also be linked to the occupations where people get exposed to contaminated environments [12].

The results indicate that flooded areas are more likely to experience cases of leptospirosis infection. Higher cases of leptospirosis were reported in Alappuzha (the most flooded district) during the post-flood phase. Additionally, other panchayats aside from the flooded panchayats also registered post-flood cases of leptospirosis. This shows that the spread of leptospirosis is not only limited to flooded areas. Other areas affected by leptospirosis could be previously endemic areas [47] or new settlements that evacuated people move into (such as relief camps) [48].

The cases in the three phases of floods were different regardless of the year involved. The lowest cases were observed during the flood phase. An evident reason for this is that the flood period was shorter in comparison with the preflood and post-flood phases. Therefore, given the incubation time of leptospirosis, infections during a flood may be reported during the post-flood phase. Additionally, the results consistently show that cases of leptospirosis are higher during the post-flood phase than in other phases [8].

Although a small part of the population was directly exposed to the flooding event, the possibility of secondary transmission cannot be ignored. Secondary transmission may be direct or indirect transmission based on the type of exposure to the pathogen. Indirect transmissions of leptospirosis are more common than direct transmission [12]. A further study is recommended to understand the influence of other risk factors of leptospirosis before and after flooding. For every 100,000 people exposed to floods, 641 were infected with the disease in the study area. This is notably higher than the global yearly rate of 14.77 cases per 100,000 estimated by the Leptospirosis Epidemiology Reference Group (LERG) [49].

This result suggests that the distribution and geographical spread of leptospirosis infections are dependent on the characteristics of floods. Nevertheless, there is a clear difference between cases in 2018 and 2019 despite floods occurring in both years in the study area: higher cases were consistently recorded after floods in Alappuzha, while only the 2018 flood led to an increase of cases in Pathanamthitta. This suggests that Alappuzha is more susceptible to flood-induced infections than Pathanamthitta. Nevertheless, a reason for the lesser number of cases in 2019 could be a result of improved awareness of leptospirosis infection which was beyond the scope of this study. Given the transmission pattern of leptospirosis, flood duration may be the most important flood severity indicator in estimating post-flood cases. *Leptospira* can survive longer in running water than in stagnant water [50]. Therefore, a longer flood event could increase the interaction between exposed people and the bacteria *Leptospira*.

The SVC regression similarly confirmed that the 2018 flood was more influential than the 2019 flood in inducing postflood cases given its statistical significance. Though previous studies have shown that leptospirosis outbreak is associated with flooding [6–8], this study further suggests that flood-induced cases are dependent on the severity of the flood event. Although floods can be a significant indicator for the prediction of future cases of leptospirosis, less severe floods may not cause a spike in the number of leptospirosis cases. Adequate forecasting and monitoring should be conducted before and during flood events to prepare for potential outbreaks in the study area. Future investigations should employ the multi-year spatiotemporal analyses used in this study to further examine the flood-leptospirosis interaction.

### Limitations of the study

This study has potential limitations due to the availability of data. The datasets describing the case and flood events are limited by incomplete data or metadata. Nevertheless, the limitations are minor and do not deter the reliability of the study's results. The quality of the flood extent data derived from radar may have been affected because of the presence of vegetation which may not have been sufficiently distinguished. In addition to this, the flood extent over the flood phase appeared to be similar in 2018 and 2019, whereas more flood problems were reported in 2018. We could not obtain flood depth data which may have provided an additional component of analysis in this study to investigate its relationship with the number of cases that occurred. Potentially inundated areas could be mapped out using novel methods [51–53].

Although the incident case data provided enough possibilities for the analyses of this study to be completed, certain limitations were encountered. A higher level of precision would have improved the spatial granularity of this research, but due to ethical and privacy concerns, the cases had to be summarized at the panchayat’s level. There is no data on the demographics of people who got the disease, this may have helped to ascertain if people in flood-related workers were at more risk than others. Additional metadata such as the date of onset of disease, the date patients were seen at the facility, laboratory test results, and mortality would have provided a better context for the analyses. There is a high chance that the cases of leptospirosis have been underreported [54], and therefore, there is an amount of uncertainty in the completeness of the epidemic data provided.

The available information about flooding events is limited by unavailable or unreliable data. The record containing the river discharge (implied by the amount of river discharge) had many missing values. This was a result of damage to station gauges at certain times. Although an attempt was made to estimate the missing values through interpolation methods by comparing the trend in other stations, the data was insufficient for a more detailed temporal analysis. In addition, the data for the flood extent obtained from satellite observations have not been verified through ground truthing.

## Conclusion

This study evaluated the incidence of leptospirosis cases in relation to flooding events by comparing the cases over flood phases and years. The total number of leptospirosis cases was higher in flooded years (2018 and 2019) than in the non-flooded year (2017). Despite the similarities between the flooding in 2018 and 2019, the 2018 flood event had a stronger impact in the study area than that of 2019. The Leptospirosis cases in both districts varied across the flood phases, but the cases were highest in the post-flood phase of both districts. The flood-induced cases of leptospirosis occurred after a time lag. While the cases in Alappuzha appeared to be similarly clustered spatially, the cases in Pathanamthitta varied significantly. Although leptospirosis is endemic in the study area, the central areas in Alappuzha were most impacted by the infectious disease due to the floods. Even though flooding led to an increase in leptospirosis cases in both years, there is stronger evidence for increased leptospirosis cases after the 2018 flood than after the 2019 flood. The significance of the 2018 flood in estimating post-flood infections could be due to the longer duration of the flood.

### Supplementary Information

Supplementary Material 1.

## Data Availability

All data used in this research are openly available and can be accessed freely except for the leptospirosis data due to its sensitive nature. Precipitation and flood information are not published but are available from the corresponding author upon reasonable request.
