# Supplementary material for: Analysing the outbreaks of leptospirosis after floods in Kerala, India
Source: Int J Health Geogr. 2024 May 13;23:11. doi: 10.1186/s12942-024-00372-9 (PMC11092194; doi:10.1186/s12942-024-00372-9)
Supplement: Supplementary file 1 — Supplementary Material 1. [file 12942_2024_372_MOESM1_ESM.docx]

### Supplementary Information

### 1 Precipitation data comparison.

The scatter plot of the values for the daily precipitation for Alappuzha in 2018 is shown in Figure 2.


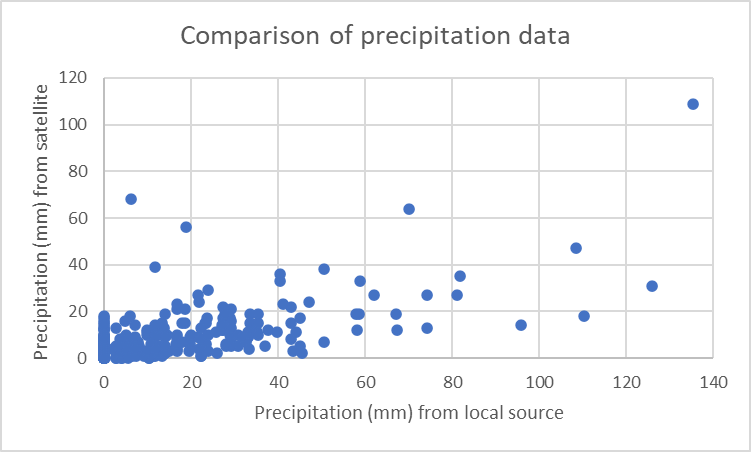


Figure 1 Precipitation from local and global sources

### 2 River discharge estimation

The summary of the missing values and the total values estimated for 2018 river discharges are provided in Table 1.

Table 1 Summary of the estimation of missing values

| Dates for which estimation was done | Number of inputted values | Dates for which estimation was done | Number of inputted values |
| --- | --- | --- | --- |
| Q_Erappuzha | | Q_Kurudamannil | |
| 16^th^ August – 20^th^ August | 5 | 25^th^ February | 1 |
| 12^th^ September – 18^th^ September | 7 | 15^th^ August – 19^th^ August | 5 |
| 26^th^ September – 28^th^ September | 3 |  |  |
| Total missing values estimated | 15 | Total missing values estimated | 6 |
| Total number of records with missing values | 193 | Total number of records with missing values | 6 |
| Total number of daily records | 365 | Total number of daily records | 365 |

### 3 Script for Regression Analysis

library(spdep)

library(maptools)

library(raster)

library(rgdal)

library(sp)

library(varycoef)

library(sf)

library(tmap)

library(gstat)

#Set working directory and import the shapefile with values for flood extent and postflood incdence

setwd("C:/MSc_Thesis/Data")

factors_HARV <- readOGR("Index", "Indicators_p2", stringsAsFactors = T)

extent(factors_HARV)

#Print the headers to confirm the file and know how to call each column

names(factors_HARV@data)

#Covert mean centre coordinates to kilometres to reduce computation time

locs <- as.matrix(factors_HARV@coords[, 1:2])/1000

fit_svc_post2 <- SVC_mle(postfl2018 ~ Flood_18, locs = locs, data = factors_HARV@data)

summary(fit_svc_post2)

fit_svc_post3 <- SVC_mle(postfl2019 ~ Flood_19, locs = locs, data = factors_HARV@data)

summary(fit_svc_post3)

### 4 Clusters and outliers for the years

The clusters and outliers of the cases of leptospirosis in 2017, 2018 and 2019 can be found in Figure 4. The clusters and outliers of the cases of leptospirosis in 2018 and 2019 can be found in Figures 5 and 6.


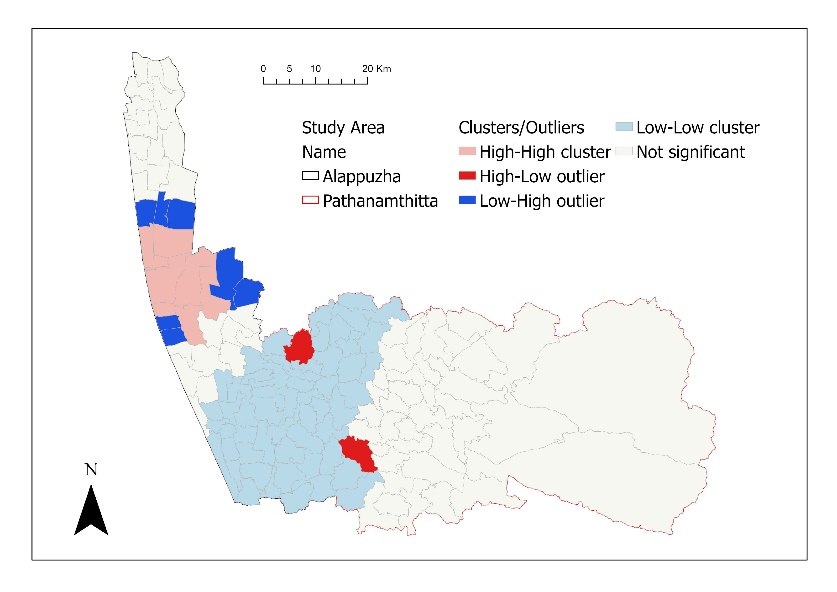

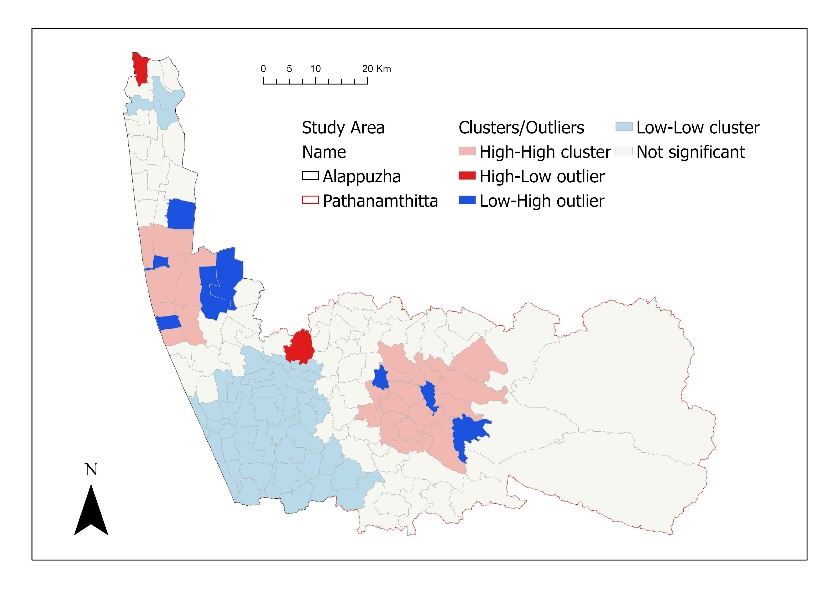

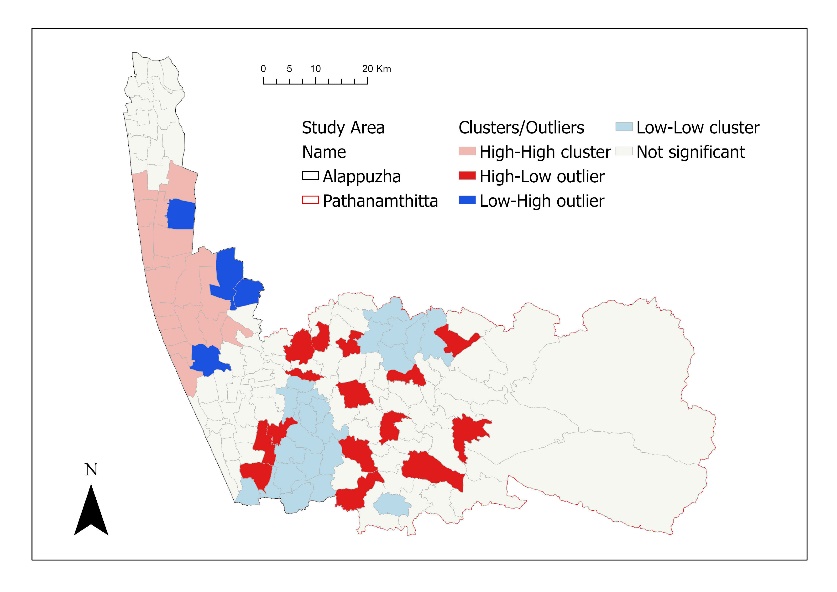


**a**

**b**

**c**

Figure 2. Cluster and outliers of cases across considered years (a) 2017 (b) 2018 (c) 2019

### 5 Clusters and outliers for the flood phases of flooded years

The clusters and outliers of the cases of leptospirosis in 2018 and 2019 during the three flood phases can be found in Figures 5 and 6.

Figure 3. Cluster and outliers of cases across flood phases (a) 2018 preflood (b) 2018 during flood (c) 2018 postflood (d) 2019 preflood (e) 2019 during flood (f) 2019 postflood


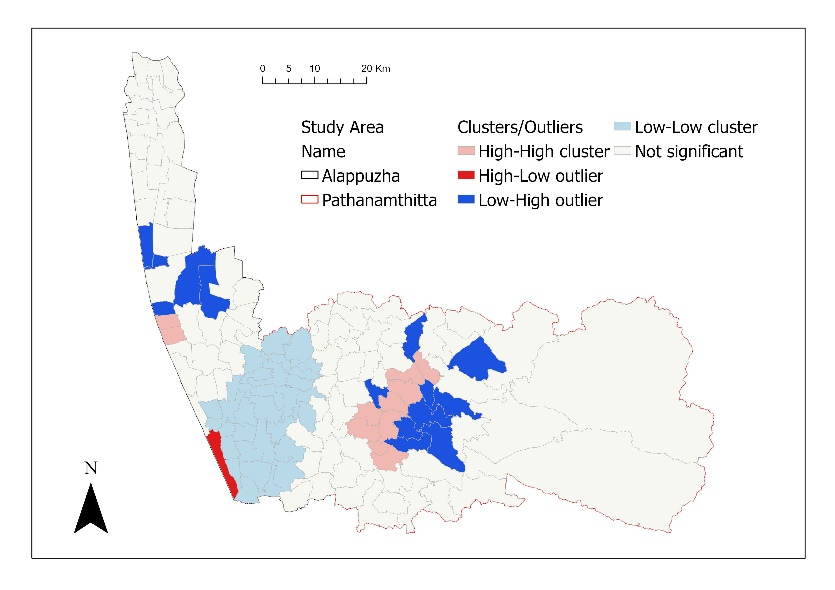

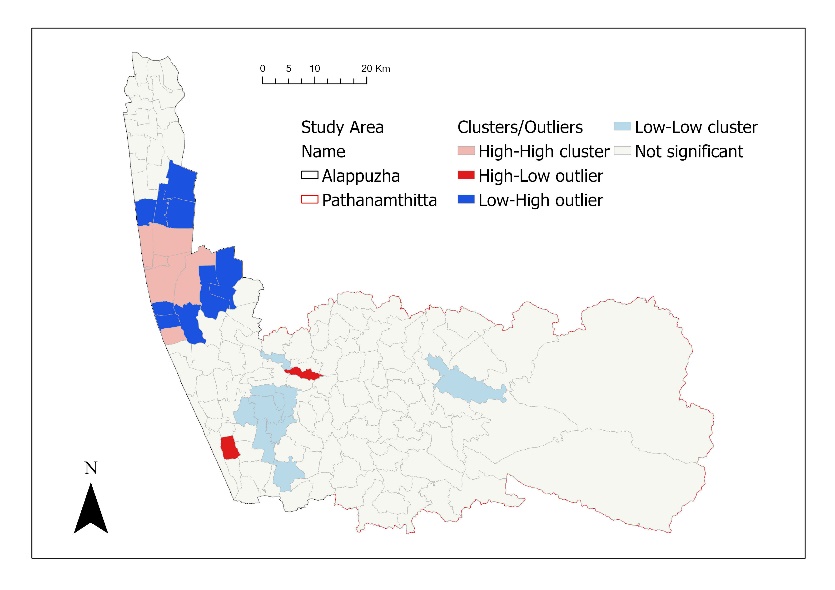

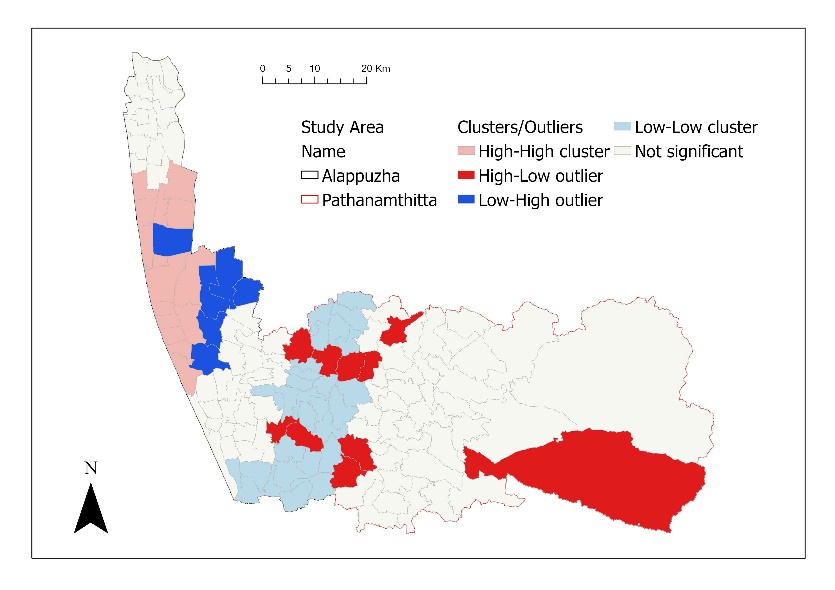


**d**

**e**

**f**


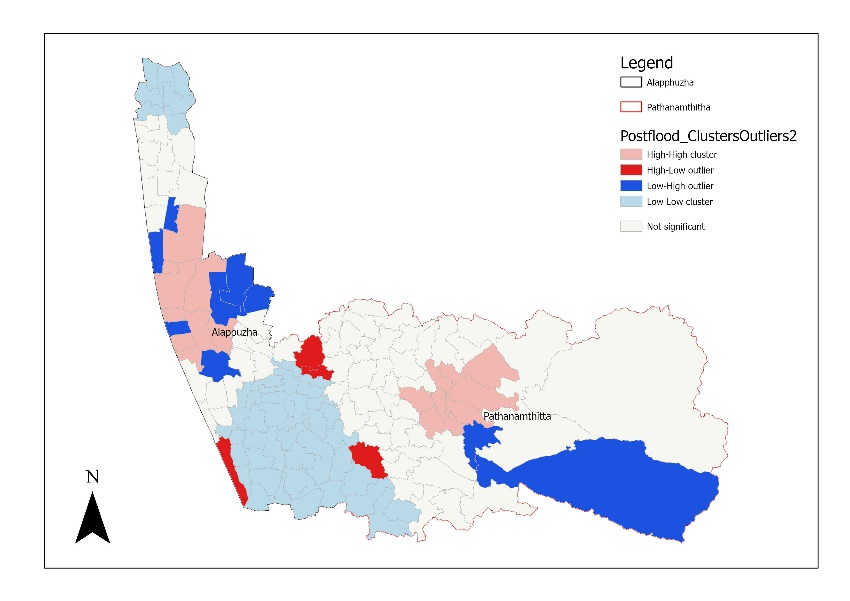

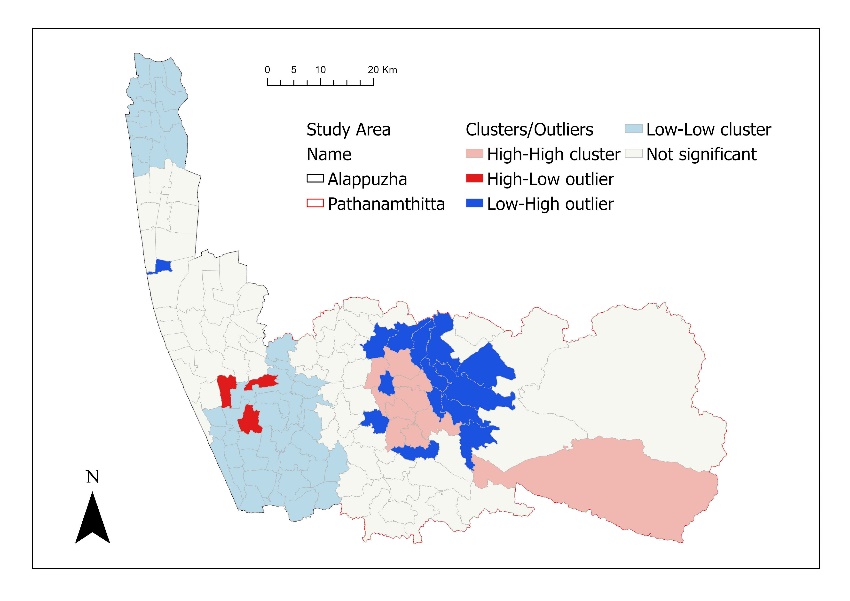

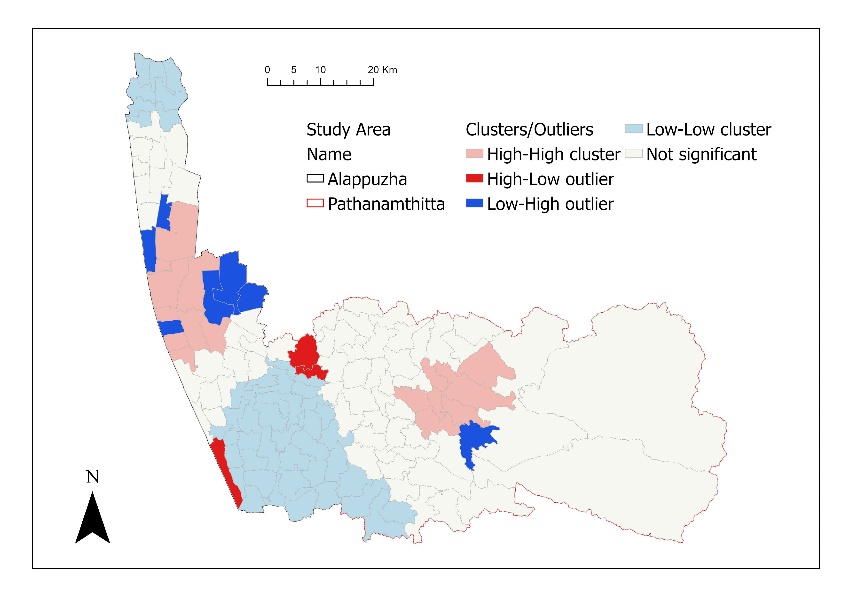


**a**

**b**

**c**
